# A Case of Respiratory Arrest After Lung Cancer Surgery due to Recurarization of Rocuronium Bromide

**DOI:** 10.1002/rcr2.70632

**Published:** 2026-05-27

**Authors:** Hiroyuki Miura, Jun Miura, Shinichi Goto, Tomoko Yamamoto

**Affiliations:** ^1^ Department of Thoracic Surgery Akiru Municipal Medical Centre Tokyo Japan; ^2^ Department of Surgery Tomei Atsugi Hospital Atsugi Kanagawa Japan; ^3^ Department of Respirology Akiru Municipal Medical Centre Tokyo Japan; ^4^ Department of Pathology Tokyo Women's Medical University Tokyo Japan

**Keywords:** lung cancer, postoperative complication, recurarization, rocuronium bromide, sugammadex

## Abstract

Sugammadex has been widely used to reverse rocuronium‐induced neuromuscular blockade. Despite apparent complete postoperative recovery, recurrence of neuromuscular blockade (recurarization) has been reported. An 82‐year‐old man with squamous cell carcinoma of the lung underwent left upper lobectomy. However, 35 min after returning to the ward, he suddenly developed respiratory arrest. Chest radiography and head computed tomography revealed no abnormalities. He recovered promptly after re‐administration of sugammadex. To our knowledge, this report has been the first to describe recurarization following lung cancer surgery. However, given that many lung cancer patients are elderly and have multiple comorbidities, the possibility of recurarization of neuromuscular blockade needs to be considered. Accordingly, a neuromuscular blockade antagonist should be readily available to manage unexpected adverse respiratory outcomes after surgery.

## Graphical abstract

An 82‐year‐old male lung cancer patient experienced respiratory arrest after surgery due to recurarization of rocuronium bromide. Considering that many lung cancer patients are elderly and have numerous comorbidities, the risk of recurarization associated with muscle relaxants should always be considered.

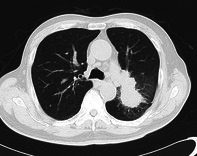

## 1 Introduction

Sugammadex has been widely used to reverse the agent for rocuronium‐induced muscle relaxation [1]. Recurrence of neuromuscular blockade (recurarization) has been reported after surgery, even among patients who appeared fully awake. This phenomenon may occur after any surgical procedure, and failure to recognize it may cause delayed or inappropriate management.

## 2 Case Report

**FIGURE 1:**
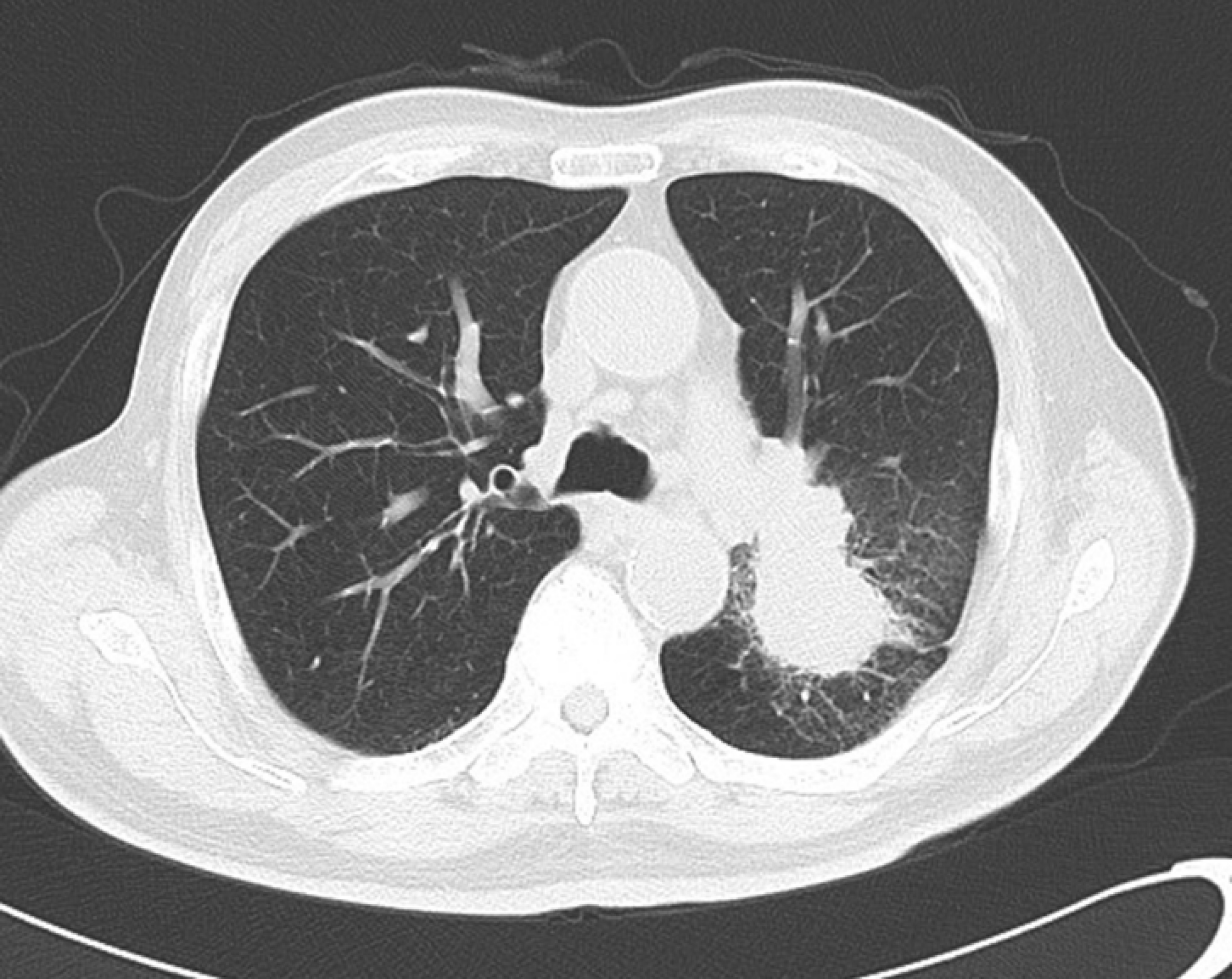
Chest computed tomography (CT) showing the tumour located in the left S1 + 2c segment with invasion into the lower lobe.

**FIGURE 2:**
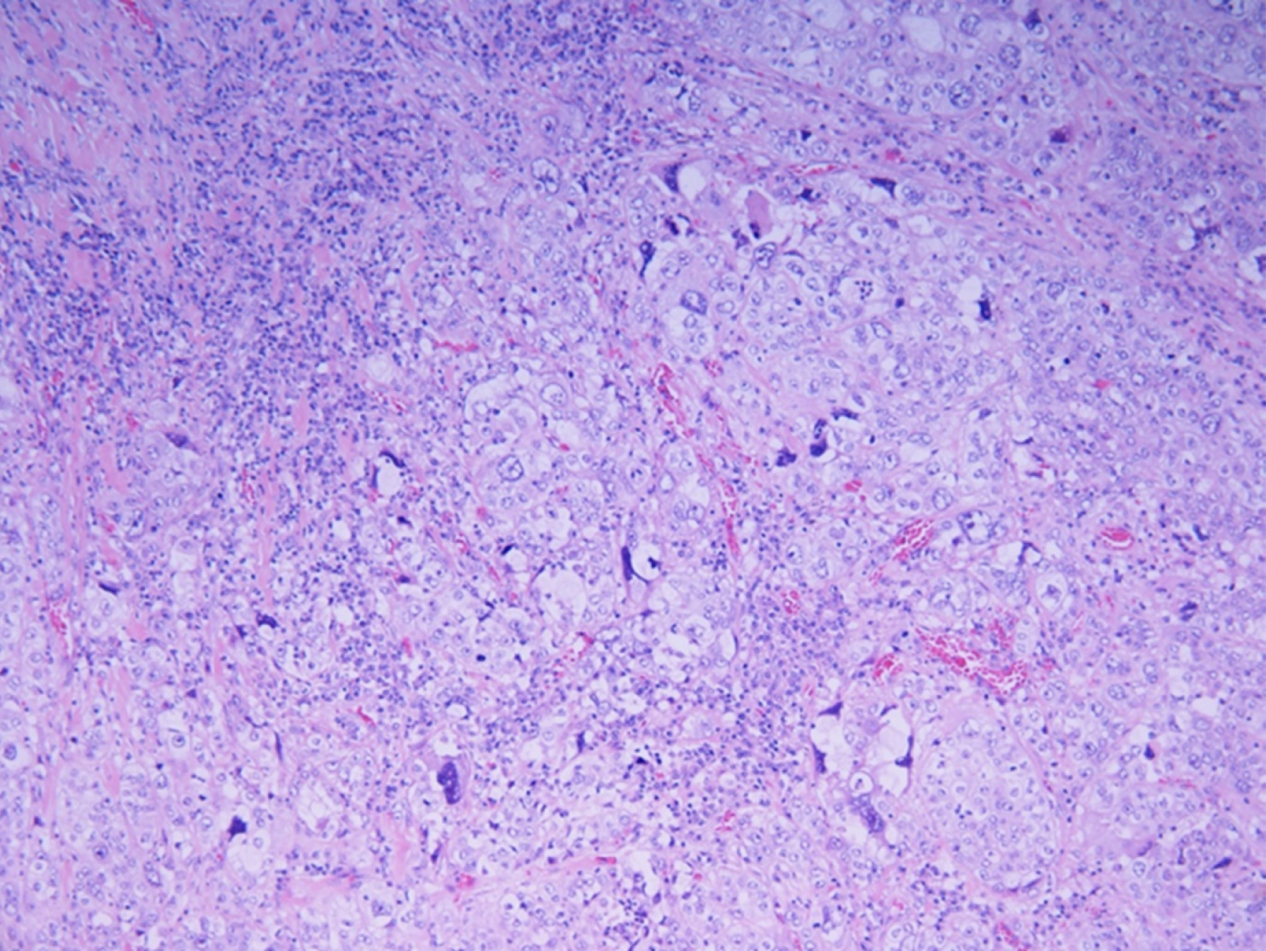
Histopathological findings demonstrating non‐keratinizing squamous cell carcinoma.

An 82‐year‐old man was referred to our hospital after an abnormal shadow was detected on an annual health check‐up. The patient had no family history of cancer but had a 53 pack‐year smoking history. Chest radiography showed an irregularly shaped mass around 38 × 27 mm in size at the left upper lung field. Chest computed tomography (CT) revealed a tumour located in the left S1 + 2c segment with associated inflammatory changes and no lymph node enlargement. Trans‐bronchial lung biopsy revealed squamous cell carcinoma. Positron emission tomography showed a maximum standardized uptake value (SUVmax) of 7.3 in the lesion, with no evidence of other abnormal uptake. The patient had restrictive ventilatory impairment with lung capacity at 78.9%. Laboratory findings revealed a white blood cell count of 14,100/μL, C‐reactive protein of 4.37 mg/dL, blood glucose of 172 mg/dL, HbA1c of 8.3% and an elevated creatinine level of 2.82 mg/dL. During blood sugar control, the tumour increased in size to 57 × 44 mm and invaded the lower lobe, with a swollen tracheobronchial lymph node (Figure 1). Left upper lobectomy was then performed after establishing a diagnosis of clinical T3N2 (single station) M0, stage IIIA disease. After confirming full recovery of consciousness and the absence of apparent muscle weakness, the patient was extubated and transferred to the ward from the operating room following careful observation. However, 35 min after returning to the ward, the patient lost consciousness and developed respiratory arrest. Endotracheal intubation was immediately performed, with no airway obstruction having been identified. No bleeding was noted from the chest tube, and a chest radiography revealed no lung collapse or blood accumulation. Head CT showed no intracranial abnormalities. The patient's blood glucose level at that time was 350 mg/dL. After considering recurarization of rocuronium bromide, sugammadex sodium was re‐administered, and the patient immediately recovered and was extubated. A total of 200 mg of sugammadex was administered to reverse the effects of the 189 mg of rocuronium bromide administered during surgery. The next day, the patient developed Mallory–Weiss syndrome but underwent endoscopic treatment and was discharged ambulatory on the eighth day after surgery. Pathological examination revealed non‐keratinizing squamous cell carcinoma measuring more than 50 mm in diameter, which was classified as stage III (pT3N1M0) (Figure 2). Although postoperative adjuvant chemotherapy was planned, treatment was delayed due to a traffic accident. During this period, brain metastases developed, and the patient died of cancer 18 months after surgery.

## 3 Discussion

Rocuronium bromide is a non‐depolarizing neuromuscular blocking agent widely used during general anaesthesia. Its onset of action is rapid, and the duration of its effect is relatively short. Meanwhile, sugammadex is a drug used to reverse rocuronium‐induced neuromuscular blockade. Inadequate dosing of sugammadex may result in recurrence of neuromuscular blockade (recurarization), a phenomenon thought to occur when unbound rocuronium redistributes from peripheral compartments back into the central and effect compartments after initial reversal [2]. The Japanese Society of Anesthesiologists had issued guidelines for monitoring the state of neuromuscular blockade before and after neuromuscular blockade. However, after the patient recovered from anaesthesia and left the operating room, the muscle relaxant monitor was detached, which makes it difficult to predict the occurrence of recurarization in the ward. Elderly patients are particularly susceptible to the prolonged effects of rocuronium, likely due to reduced cardiac output and clearance caused by impaired cardiac, hepatic and renal function. Given that rocuronium is excreted renally, its excretion may be prolonged in patients with renal impairment. Although this case occurred before the publication of current guidelines, recurarization still occurred despite confirmation of full consciousness. Hence, recurarization in the current case may have been associated with advanced age and renal dysfunction secondary to diabetes mellitus. Elderly patients with impaired renal function are not uncommon. It is worth noting that recurarization can occur in any patient. Furthermore, among obese patients [3], calculating the dose of the drug based on their actual body weight may result in overdosing. Similarly, gestational weight gain and increased adipose tissue may increase the total administered dose compared to non‐pregnant patients, potentially prolonging the drug's effects. Moreover, hypothermia delays excretion into urine and bile, potentially prolonging the drug's effects, highlighting the need for caution during hypothermic anaesthesia [4]. Patients with myasthenia gravis are highly sensitive to non‐depolarizing neuromuscular blocking agents and require careful dose adjustment and monitoring [5]. Recurarization has also been reported in amyotrophic lateral sclerosis.

Recurarization after general anaesthesia is reported throughout the context of gastrointestinal cancer surgery, nephrectomy in urology and femoral fracture surgery in orthopaedics, but in all cases, renal dysfunction or obesity was observed. In other words, it is a complication that can occur in any surgery. To our knowledge, this report has been the first to describe a case of respiratory arrest due to recurarization following lung cancer surgery. Given that many patients undergoing lung cancer surgery are elderly and have multiple comorbidities, clinicians should remain vigilant of the potential risk of recurarization. In elderly patients, especially those with renal dysfunction, frequent observation and monitoring are crucial, keeping in mind the possibility of recurarization after general anaesthesia. It is also essential to ensure that the emergency cart is well‐stocked and checked so that a prompt response can be given in the event of respiratory arrest. Moreover, antagonists must be available to treat postoperative respiratory arrest.

## Author Contributions

Dr. Hiroyuki Miura and Dr. Shinichi Goto helped in the conception and design of the study and analysis or interpretation of data for the work. Dr. Jun Miura drafted the study and revised it critically for important intellectual content. Dr. Yamamoto diagnosed this cancer pathologically. All authors contributed to the final version of this manuscript and approved it to be published.

## Funding

The authors have nothing to report.

## Consent

The authors declare that written informed consent was obtained for the publication of this manuscript and accompanying images using the form provided by the Journal.

## Conflicts of Interest

The authors declare no conflicts of interest.

## Data Availability

The data that support the findings of this study are available on request from the corresponding author. The data are not publicly available due to privacy or ethical restrictions.
